# MALT1 is a potential therapeutic target in glioblastoma and plays a crucial role in EGFR‐induced NF‐κB activation

**DOI:** 10.1111/jcmm.15383

**Published:** 2020-05-25

**Authors:** Xuejiao Liu, Chenglong Yue, Lin Shi, Guanzheng Liu, Qiyu Cao, Qianqian Shan, Yifeng Wang, Xiangyu Chen, Huan Li, Jie Wang, Shangfeng Gao, Mingshan Niu, Rutong Yu

**Affiliations:** ^1^ Insititute of Nervous System Diseases Xuzhou Medical University Xuzhou China; ^2^ Department of Neurosurgery the Affiliated Hospital of Xuzhou Medical University Xuzhou China; ^3^ Surgical Deparment 9 Xuzhou Children's Hospital Xuzhou China; ^4^ Blood Diseases Institute Xuzhou Medical University Xuzhou China

**Keywords:** cell proliferation, EGFR/ NF‐κB signalling pathway, GBM, MALT1, MI‐2

## Abstract

Glioblastoma multiforme (GBM) is the most common malignant tumour in the adult brain and hard to treat. Nuclear factor κB (NF‐κB) signalling has a crucial role in the tumorigenesis of GBM. EGFR signalling is an important driver of NF‐κB activation in GBM; however, the correlation between EGFR and the NF‐κB pathway remains unclear. In this study, we investigated the role of mucosa‐associated lymphoma antigen 1 (MALT1) in glioma progression and evaluated the anti‐tumour activity and effectiveness of MI‐2, a MALT1 inhibitor in a pre‐clinical GBM model. We identified a paracaspase MALT1 that is involved in EGFR‐induced NF‐kB activation in GBM. MALT1 deficiency or inhibition significantly affected the proliferation, survival, migration and invasion of GBM cells both in vitro and in vivo. Moreover, MALT1 inhibition caused G1 cell cycle arrest by regulating multiple cell cycle–associated proteins. Mechanistically, MALTI inhibition blocks the degradation of IκBα and prevents the nuclear accumulation of the NF‐κB p65 subunit in GBM cells. This study found that MALT1, a key signal transduction cascade, can mediate EGFR‐induced NF‐kB activation in GBM and may be potentially used as a novel therapeutic target for GBM.

## 1 INTRODUCTION

Glioblastoma multiforme (GBM) is the highest grade of malignant glioma and the most common and lethal type of brain tumour in adults.^1^, ^2^, ^3^ Advances in neurosurgery, radiotherapy and chemotherapy have facilitated in the improvement of treatment schemes for GBM; however, the prognosis of patients with GBM remains dismal, with a median survival of 12 to 15 months after initial diagnosis.^4^, ^5^ Therefore, there is a need for a detailed investigation on the clinical pathogenesis of GBM to discover novel molecular targets for treatment and to improve prognosis.

Nuclear factor κB (NF‐κB) activation commonly occurs in cancer, and substantial experimental evidence suggests that it is involved in both cancer development and resistance to treatment.^6^, ^7^, ^8^ Previous studies have reported that NF‐κB activation is a core driver for the malignant phenotype of GBM, which is correlated to a negative prognosis in patients with GBM.^9^, ^10^ Constitutive NF‐κB activation promotes the expression of various proteins involved in the proliferation, survival, migration and epithelial‐to‐mesenchymal transition of GBM cells.^11^ However, different types of cancers activate NF‐κB using different mechanisms. Hence, uncovering signalling pathways that lead to activation of NF‐κB in GBM will provide the therapeutic targets and thus benefit tumour treatment.

Epidermal growth factor receptor (EGFR) signalling activates NF‐κB because EGFR gene amplification and mutation are common in GBM, and the aberrant EGFR signalling is likely to be a vital mechanism of NF‐kB activation in GBM.^12^, ^13^, ^14^ Although many reports confirm that EGFR signalling is an important driver of NF‐κB activation in GBM, the underlying molecular mechanism remains largely unknown. Elucidating the association of EGFR with the NF‐κB pathway in GBM will thus provide novel drug targets for the inhibition of GBM progression. Therefore, the identification of a specific signalling component with therapeutic potential and high specificity in the tumour‐promoting NF‐κB pathway may help improve therapeutic efficacy.

Mucosa‐associated lymphoma antigen 1 (MALT1) is a paracaspase that belongs to the caspase family of proteases and possesses arginine‐specific cysteine protease activity.^15^ MALT1 mediates by proteolytic cleavage that inactivates inhibitors of the NF‐κB signalling pathway such as TNFAIP3/A20, and the proteins that favour NF‐κB activity such as CYLD and RelB.^16^ In addition, MALT1 serves as a scaffold to recruit additional signal transducers to activate NF‐κB signalling.^17^ Pan et al have reported that MALT1 is involved in EGFR‐induced NF‐κB activation and promotes EGFR‐associated solid tumour progression.^18^ Moreover, other researchers also found that miR‐181d could attenuate the mesenchymal phenotype by directly repressing MALT1 in glioblastoma.^19^ Thus, MALT1 may be considered as a potential target of solid tumour treatment. Recently, Fontan and co‐authors discovered a novel molecule called MI‐2 that could inhibit MALT1 by forming a covalent linkage in the active site.^20^ Therefore, MI‐2 can be used as a therapeutic agent for the treatment of tumours that are dependent on MALT1 signals.

In the present study, we identified MI‐2 that effectively inhibited the activity of NF‐κB in GBM by inhibitor screening. Then, we investigated the mechanism and therapeutic potential of MALT1 inhibition in the treatment of GBM. The results show that MALT1 is required for EGFR‐induced NF‐kB activation in GBM cells. Our study provides insights into the applicability of MALT1 as a potential therapeutic target for GBM and describes the basis for further clinical investigations of MI‐2 in GBM.

## 2 MATERIALS AND METHODS

### 2.1 Cell lines and antibodies

Human GBM cell lines U251 and U87 were purchased from the Shanghai Cell Bank, Chinese Academy of Sciences for this study. These cell lines were cultured in DMEM supplemented with 10% FBS at 37°C in a humidified incubator with 5% CO_2_. Antibodies against MALT1, p‐pRb, cyclin D1, CDK4, p27, p21, p65, IκB‐α and lamin A/C were obtained from Cell Signaling Technology (CST, Beverly, MA, USA). Antibodies specific to β‐actin were purchased from Abcam (Cambridge, MA, USA). DAPI was obtained from Sigma‐Aldrich Chemical Co. (St. Louis, MO, USA).

### 2.2 Construction and production of the lentivirus

The CRISPR/Cas9 system was used to knock out MALT1 in GBM cells. A non‐targeting sgRNA was used as control. We used the two MALT1 sgRNAs targeting the human *MALT1* gene to generate MALT1‐knockout cells as previously described.^21^ The sequences of the sgRNA used in this study are as follows: control‐sgRNA, 5'‐TACTAACGCCGCTCCTACAG‐3'; MALT1‐sgRNA, 5′‐GCAGTGCATGTAAAAGATGC‐3′; and MALT1‐sgRNA2, 5′‐ATTCAGCCAGTGGTCACAGC‐3’. The viruses were propagated in 293T cells by co‐transfecting the corresponding plasmids with the helper plasmids pSPXA2 and pMD2.G with Lipofectamine 2000 (Invitrogen, Carlsbad, CA, USA). The supernatant was collected and concentrated by ultracentrifugation after 48 hours of incubation.

### 2.3 Establishment of stable cell lines

MALT1‐sgRNA and control lentivirus were used to transfect GBM cell lines for 72 hours, and the transfected cells were cultivated with 2.5 μg/mL puromycin. The stable cell lines were obtained by cultivating the surviving cells, and the MALT1‐knockout cell lines were named as MALT1‐sgRNA and MALT1‐sgRNA2. DNA was isolated from the constructed cell lines, and the PCR amplification products were collected for sequencing.

### 2.4 Cell viability assay

Cell counting kit‐8 (CCK‐8, Dojindo, Japan) was used for evaluating cell viability by cultivating in triplicate 96‐well culture plates (3000 cells/well) and incubated at 37°C for 24, 48, 72 and 96 hours. Then, 10 μL CCK‐8 solution was added to each well and cultured for 3 hours before measuring absorbance at a wavelength of 450 nm using a microplate reader.

### 2.5 EdU incorporation assays

A Cell‐Light™ EdU Cell Proliferation Detection Kit was used to investigate cell proliferation. Briefly, cells were seeded into 96‐well plates and incubated overnight, treated with MI‐2 at different concentrations (0, 2 and 4 μmol/L) for 12 hours, incubated with 50 μmol/L EdU for 4 hours and then fixed using 4% paraformaldehyde for 30 minutes. Thereafter, the cells were treated with 0.5% Triton X‐100 for 20 minutes and then incubated with 100 μL of 1× Apollo^®^ reaction cocktail for 30 minutes. Cellular DNA was stained with DAPI for 15 minutes and washed with PBS thrice. Finally, the cells were analysed using a fluorescence microscope (Olympus, Japan), and images were captured.

### 2.6 Clonogenic cell survival assay

Cells were treated with 0.1% DMSO (vehicle) or MI‐2 (0‐4 μmol/L) in six‐well plates (500 cells/well) for 12 hours. The culture medium in each well was replaced every 4 days during the 10‐14 days of colony formation. Then, the cells were fixed with 4% formaldehyde and stained with 0.1% crystal violet solution. Positive colony formation was counted manually.

### 2.7 Cell cycle assay

To determine cell cycle distribution after MALT1 knockout or MI‐2 treatment, cell cycle analysis was conducted as previously described.^22^, ^23^ Briefly, the cells were seeded into six‐well plates at a density of 2 × 10^6^ cells per well and treated with MI‐2 (0‐4 μmol/L) for 24 hours. After treatment, both floating and attached cells were collected, fixed in 70% ethanol, washed twice with PBS and then stained with PI solution that contained 50 µg/mL PI and 25 µg/mL RNase in the dark for 30 minutes. Then, the treated cells were assessed on a FACSCalibur (Becton‐Dickinson, Franklin Lakes, NJ, USA) using CellQuest Pro software (Becton‐Dickinson, Franklin Lakes, NJ, USA).

### 2.8 Wound‐healing assay

Cells were obtained with a plastic pipette tip and washed with PBS to remove debris. Then, the cells were incubated and treated with serum‐free media with or without MI‐2. Three different time regimens (0, 24 and 48 hours) and five selected points of the lesions were assessed using a microscope (Olympus, Japan).

### 2.9 Invasion assay

A Transwell^®^ system was used to test cell invasion in vitro as previously described.^5^, ^24^ Briefly, cells from MI‐2–treated and control group were applied on the upper chambers with 8‐μm pore polycarbonate filters pre‐coated with Matrigel^®^; the lower chamber was filled with DMEM supplemented with 10% FBS as chemoattractant. After incubating at 37°C for 30 hours, the cells were then transferred to the bottom region of the filter membrane, fixed with 4% paraformaldehyde for 30 minutes and then stained with 0.1% crystal violet solution for 30 minutes. Invading cells from five randomly selected points were imaged and counted under an inverted microscope (Olympus, Japan).

### 2.10 Western blot analysis

The expression level of proteins was examined by Western blot analysis according to previous reports.^25^, ^26^ Total protein and cytoplasmic protein were extracted from the MALT1‐knockout cells and MI‐2–treated cells, and the concentrations of these proteins were measured using BCA Protein Assay Kit (Beyotime, China). Electrophoresis (10% SDS‐PAGE gel) was used to separate these proteins (50 μg/lane) in each sample and then transferred onto polyvinylidene difluoride (PVDF) membranes for further analysis. The membrane was blocked with 5% non‐fat milk and probed with specific primary antibodies at 4°C overnight, and secondary antibodies were added at room temperature for 2 hours. Then, these proteins were detected using an enhanced chemiluminescence detection system.

### 2.11 Ethical statement

A total of 74 mice (male, BALB/c nu/nu, 5‐6 weeks, 18‐20 g) used in this study were purchased from Weitong Lihua Experimental Animal Technology Co., Ltd. (No. SCXK 2016‐0006, Beijing, PR China). The mice were allowed to acclimatize for seven days. The mice were housed on a 12‐hour light, 12‐hour dark cycle with free access to food and water. Animal procedures were conducted according to the Guidelines for the Care and Use of Laboratory Animals (8th Ed., National Institutes of Health). All the experiments were done with ethics committee approval and met the requirements of Xuzhou Medical University.

### 2.12 In vivo experiments

MALT1‐knockout U87 (5 × 10^5^ cells per mouse) cells and control cells were stereotactically implanted into the right striata of nude mice as previously described.^27^, ^28^ The animals used were randomly assigned. Sixteen mice were assigned in each group. Five of the mice were used to evaluate tumour size, and another five were used to assess the survival analysis. To test the efficiency of MI‐2 treatment, U87 cells (5 × 10^5^ cells per mouse) were injected into the brain of nude mice. Seven days later, the treated mice (n = 42) were randomly divided into three different groups: MI‐2 with 20 mg/kg (n = 14), MI‐2 with 40 mg/kg (n = 14) and vehicle (n = 14). The drugs and vehicle were delivered daily via intraperitoneal injections. The mice were killed when cachexia occurred, and their brains were removed, fixed with 4% paraformaldehyde and dehydrated sequentially with 20% and 30% sucrose at 4°C until sinking. For Kaplan‐Meier survival analysis, the mice (n = 9 per cohort) were killed when these exhibited neurological symptoms that included weight loss, mental apathy and haemiplegia.

### 2.13 Histopathology and immunofluorescence staining

The flash‐frozen brains were serially cut at 12 μm thicknesses, and brain section that contained the largest tumour area was stained with haematoxylin and eosin (H&E) as previously described.^28^ Proliferative and apoptotic indices of the tumours were assessed by immunofluorescence staining of anti‐Ki67 and anti‐cleaved caspase‐3. Briefly, the brain sections with the tumour were incubated in 0.1% Triton X‐100 and blocked with 1% BSA in PBS for 1 hour. Subsequently, primary and secondary antibodies were applied to the brain slices and incubated. Cellular DNA was stained with DAPI for 15 minutes. All brain slices were examined and photographed with a microscope with an attached fluorescence detector. We used three tumour tissue sections for Ki67 and cleaved caspase‐3 quantitative analysis. Ki67‐ and cleaved caspase‐3–positive cells were counted in five randomly selected areas. Signal intensity was measured with ImageJ software.

### 2.14 Statistical analyses

The data obtained in this study were processed with GraphPad Prism 6 software. One‐way ANOVA and Student's *t* test were applied to assess statistical differences among groups, and survival analysis was evaluated by Kaplan‐Meier survival curve and the log‐rank test. *P*‐values were marked as *P < 0.05, **P < 0.01 and ***P < 0.001.

## 3 RESULTS

### 3.1 MALT1 contributes to GBM cell proliferation and colony formation

**FIGURE 1:**
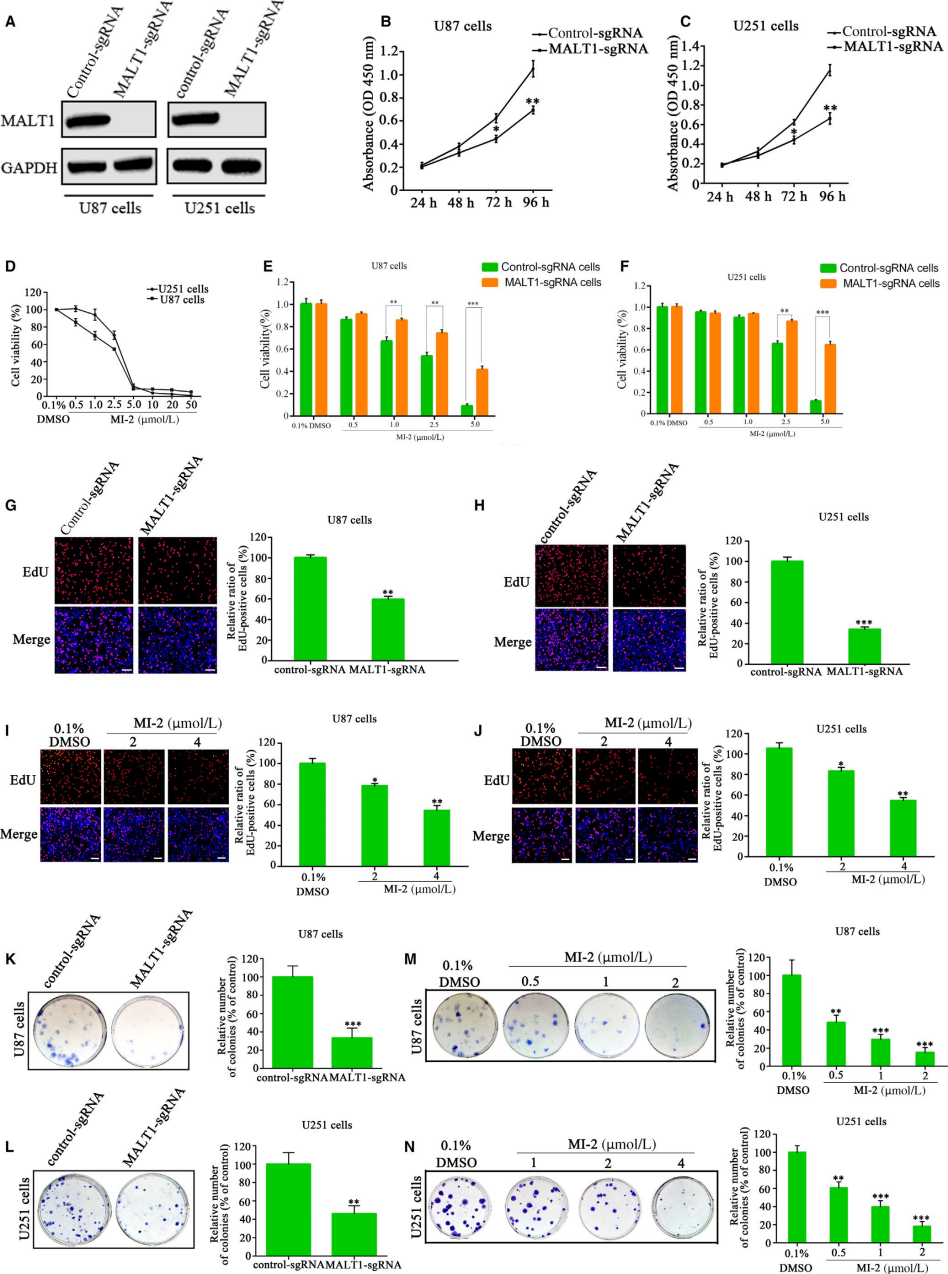
MALT1 inhibition suppresses the proliferation of GBM cells. A, Knockout efficiency of MALT1 was examined by Western blot analysis in MALT1‐sgRNA and corresponding control cells. B‐D, CCK8 assays were used to measure the cell viability by knocking out MALT1 or using MALT1 inhibitor MI‐2. E‐F, Cell viabilities were assessed in MALT1 knockout and control cells with MI‐2 treatment (0‐5 μmol/L) by CCK‐8 assays. G‐H, Measurement of anti‐proliferation effects of MALT1 knockout by EdU incorporation assay, *scale bar*: 100 μm. I‐J, Representative images from EdU analysis of cell proliferation after treatment of U87 and U251 cells with MI‐2, *scale bar*: 100 μm. K‐L, Cell proliferation ability after MALT1 knockout was examined by colony formation assay. M‐N, MI‐2 inhibits the colony formation of U87 and U251 cells. The number of proliferative cells or colony formation was normalized to that of the control group. The data are presented as mean ± SEM of three replicates, *P < 0.05, **P < 0.01, ***P < 0.001

Western blot analysis was used to test the efficiency of U87 and U251 cell lines with MALT1 knocked out. Figure 1A clearly illustrates that the MALT1‐knockout group did not express MALT1 compared with the sgRNA‐negative control group, which means the MALT1 was successfully knocked out by MALT1‐sgRNA. The sequencing results are shown in Figure S1 of Supplemental materials. CCK‐8, EdU and colony formation assays were performed to assess the effects of MALT1 on cell proliferation. Figure 1B–C show that cell numbers decreased by 42.78% in MALT1‐sgRNA U87 and 34.03% in MALT1‐sgRNA U251 cells after 96 hours of incubation. Similarly, the number of EdU‐positive cells sharply decreased in the MALT1‐sgRNA group compared with the control (Figure 1G,H). The clonogenic potential of the U251 and U87 cells was inhibited after MALT1 knockout (Figure 1K,L). The average number of colonies formed decreased to 33.33% and 46.05% in the U87 and U251 cells compared with the control. To exclude the potential off‐target effect, we also used another sgRNA to knock out *MALT1* gene. We observed a similar inhibitory effect on GBM cells when MALT1 was knocked out by MALT1‐sgRNA2 (Figure S2). Taken together, the results clearly demonstrate that MALT1 effectively promotes the proliferation of GBM cells.

MALT1 inhibitor MI‐2 was used to examine GBM cell growth to assess its role in the pathogenesis of GBM. The results showed that MI‐2 dose‐dependently reduced the viability of the U251 and U87 cells (Figure 1D). To further investigate whether the effect of MI‐2 is specific to MALT1 protease activity, MALT1‐deficient and corresponding control cells were treated with MI‐2 for 72 hours, and then, CCK‐8 assays were used to assess cell viability. The results showed that MI‐2 treatment significantly inhibited the growth of cells expressing MALT1. However, the inhibitory effects of MI‐2 on the growth of glioma cells were reduced in the MALT1‐deficient cells (Figure 1E,F). These findings indicate that MI‐2 is specific to MALT1 protease activity. Similarly, the inhibitory efficiency of MI‐2 on the proliferation and colony formation ability of GBM cells was detected by EdU incorporation and colony formation assays. The results showed that MI‐2 could significantly inhibit cell proliferation and colony formation of GBM cells (Figure 1I,J,M,N). This evidence confirms the vital impact of MALT1 on GBM cell growth.

### 3.2 MALT1 inhibition induces G1 cell cycle arrest in GBM cells

**FIGURE 2:**
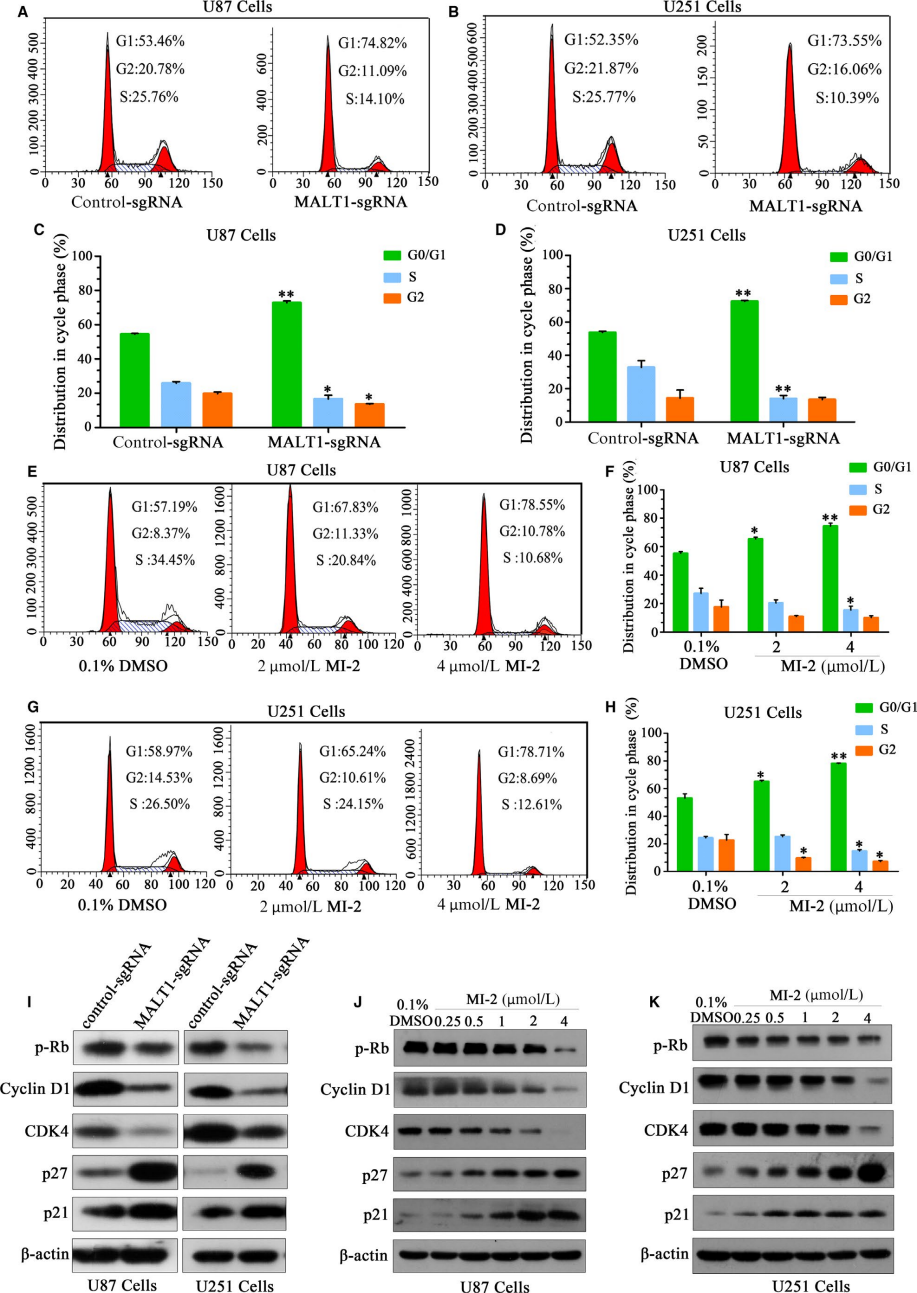
The effects of MALI1 knockout or inhibition by MI‐2 on cell cycle progression in GBM cells. A‐D, The distribution of cell cycle and quantitative analysis after MALT1 knockout in U87 and U251 cells was examined by flow cytometry. E‐H, Representative data from the cell cycle analysis of MI‐2–treated cells. Quantitative analysis of cycle phase distribution in the control and MI‐2–treated groups. I, MALT1 knockout regulated the expression of cell cycle–related proteins. J‐K, MI‐2 treatment affected the expression levels of cell cycle–related protein levels. The data are presented as the mean ± SEM of three replicates, *P < 0.05, **P < 0.01

Cell proliferation is linked to cell cycle progression. To adjudge the effect of MALT1 inhibition on this connection, flow cytometry with propidium iodide staining was used to assess the influence of MALT1 knockout or MI‐2 treatment on cell phase distribution. The results showed a higher proportion of U87 MALT1‐knockout cells were in the G1 phase (74.82%) than the control‐sgRNA cells (53.46%). Similar results were observed in MALT1‐knockout U251 cells (Figure 2A‐D). In contrast, knocking out MALT1 led to a marked decrease compared with the control cells in S phase. Moreover, the two cell lines (U87 and U251) were arrested at the G1 phase of the cell cycle after treatment with MI‐2 (Figure 2E‐H). These results suggest that inhibition of MALT1 expression results in the arrest of GBM cells at the G1 phase.

Western blot was also used to evaluate the effect of MALT1 knockout or MI‐2 treatment on the expression level of cell cycle regulating factors. The expression levels of the inhibited proteins p27 and p21 were strongly enhanced after knocking out MALT1 (Figure 2I). In contrast, the expression levels of pRb, cyclin D1 and CDK4 in the MALT1‐knockout cells markedly decreased compared with the controls (Figure 2I). The similar trends were observed in MI‐2–treated cells (Figure 2J‐K). These results verify that inhibited MALT1 leads to G1 arrest of GBM cells that is caused by altering various cell cycle regulatory proteins.

### 3.3 MI‐2 inhibits the migration and invasion of GBM cells

**FIGURE 3:**
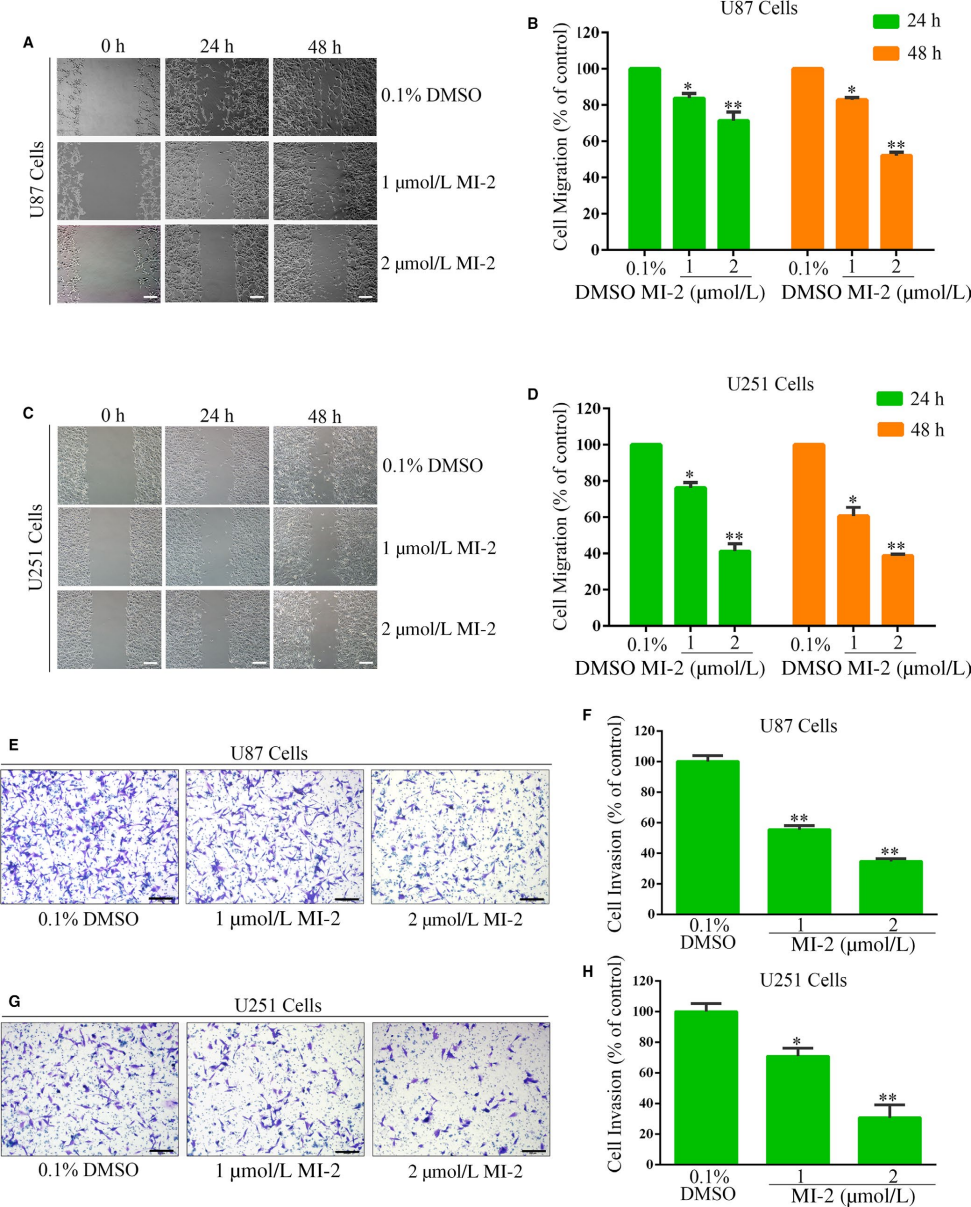
MI‐2 inhibits the migration and invasion of GBM cells. (A and C) The effect of MI‐2 on U87 and U251 cell migration was examined using a wound‐healing assay. (B and D) Quantitative analysis of migratory cell numbers. (E and G) Cell migratory ability after MI‐2 treatment was assessed using a Transwell^®^ invasion assay. (F and H) Assessment of the number of cells that invaded the filter. The number of migratory or invading cells was normalized to the control group. The data are presented as the mean ± SEM of three replicates, *P < 0.05, **P < 0.01, *scale bar*: 200 μm

Wound‐healing assay was performed to assess the role of MALT1 in the migration of GBM cells. The migration of U87 and U251 cells into scratch wounds is shown in Figure 3A,C, which indicate that the number of cells that had migrated into the wound decreased via a concentration‐dependent way. Figure 3B illustrates that the number of U87 cells that migrated, respectively, decreased to 71.63% and 52.65% 24 and 48 hours after 2 μmol/L MI‐2 treatment. A striking loss of migratory U251 cells was observed at 24 and 48 hours, with the respective reductions of 57.62% and 62.82% after treatment with 2 μmol/L MI‐2.

The Transwell^®^ invasion assay was performed to investigate the effect of MI‐2 on the invasion of GBM cells by treating U87 and U251 cells with MI‐2 for 30 hours. The number of invasive MI‐2 treated U87 cells decreased to 55.47% and 34.67% at 1 and 2 μmol/L, respectively (Figure 3E,F). Similar trends were observed in the U251 cells, which exhibited a 26.61% and 62.36% decrease at 1 and 2 μmol/L, respectively (Figure 3G,H). This phenomenon suggests that the migration and invasion of GBM cells can be remarkably reduced by MI‐2, and we hypothesize that MALT1 controls GBM cells migration and invasion.

### 3.4 Knocking out MALT1 or inhibition by MI‐2 suppresses intracranial GBM growth in vivo

**FIGURE 4:**
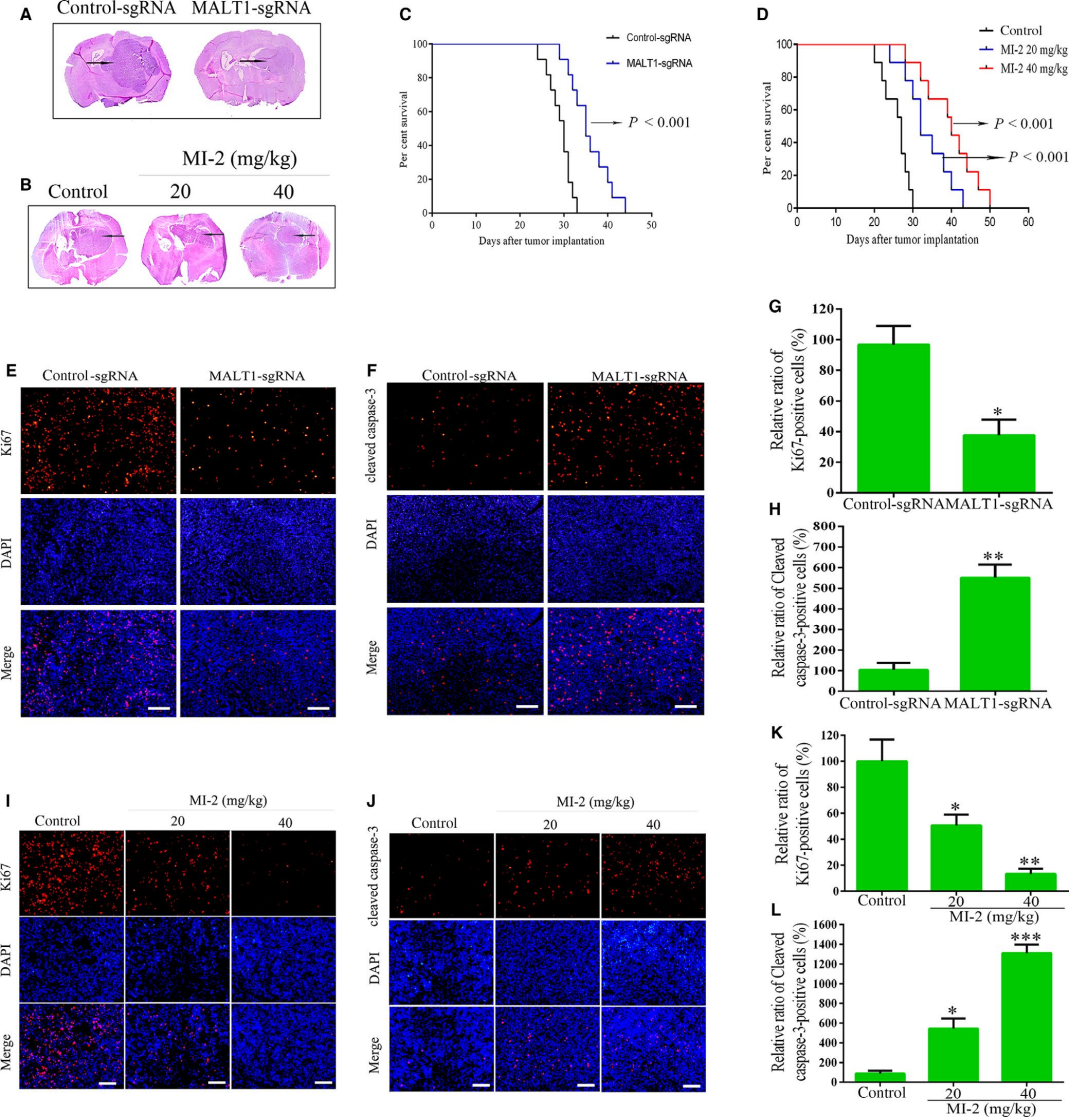
MALT1 knockout or inhibition suppresses intracranial GBM growth in vivo. A, Representative images of HE staining of tumours derived from control and MALT1‐knockout nude mice. B, Brain tissues were dissected and then stained with H&E to evaluate tumour formation after MI‐2 treatment. C, Kaplan‐Meier curve of control and MALT1‐knockout mice showed a clear survival advantage for MALT1 knockout (n = 11; *P* < 0.001, determined using log‐rank test). D, The survival of mice with tumours derived from the vehicle or MI‐2–treated groups was assessed using Kaplan‐Meier survival curves. E‐L, Tumour cell proliferation and apoptosis were assessed with anti‐Ki67 and anti‐cleaved caspase‐3 immunostaining. Quantitative analysis of the percentage of positive cells in the MALT1‐knockout or MI‐2‐treated groups that were normalized to the control group. **P < 0.01, ***P < 0.001, *scale bar*: 250 μm

To examine whether MALT1 is important to GBM growth in vivo, MALT1‐knockout U87 cells were transplanted into the right striatum of nude mice using a stereotactic technique. H&E staining (Figure 4A) showed that tumours derived from MALT1‐knockout U87 cells were significantly smaller than those of the control group. Furthermore, the median survival time was prolonged after MALT1 knockout (Figure 4C), indicating that MALT1‐knockout mice had a clear survival advantage.

Immunostained tumour tissues isolated from control and MALT1‐knockout mice were used to evaluate the effect of MALT1 knockout on cell proliferation and apoptosis in vivo. Compared with the control, a lower number of Ki67‐positive tumour cells were observed in the MALT1‐knockout mice. However, the proportion of apoptotic cells showed a 5.5‐fold increase in the MALT1‐knockout group (Figure 4E‐H). This phenomenon clearly indicated that knocking out MALT1 disrupts tumour cell proliferation and causes cell apoptosis in vivo.

Furthermore, we evaluate the impact of MI‐2 on the anti‐tumour activity of glioblastoma cells in vivo. Figure 4B shows that compared with vehicle‐treated mice, MI‐2 treatment significantly suppressed xenograft tumorigenesis and reduced the size of the tumours. The survival of nude mouse bearing U87 cells improved with MI‐2 treatment (Figure 4D). Compared with the control group, no significant change in weight was observed in the MI‐2–treated group (data not shown). In addition, we also found that the mean percentages of Ki67‐positive cells significantly decreased to 50.59% and 13.36% in the 20 and 40 mg/kg MI‐2–treated groups, respectively (Figure 4I,K). In contrast, a striking increase in cleaved caspase‐3–positive cells was observed in the 20 and 40 mg/kg MI‐2–treated groups, which showed a 5.4‐ and 13.1‐fold increase, respectively (Figure 4J,L). In summary, these data reveal that MI‐2 can inhibit tumour cell growth in vivo.

### 3.5 MALT1 is required for EGF‐induced NF‐κB activation in GBM cells

**FIGURE 5:**
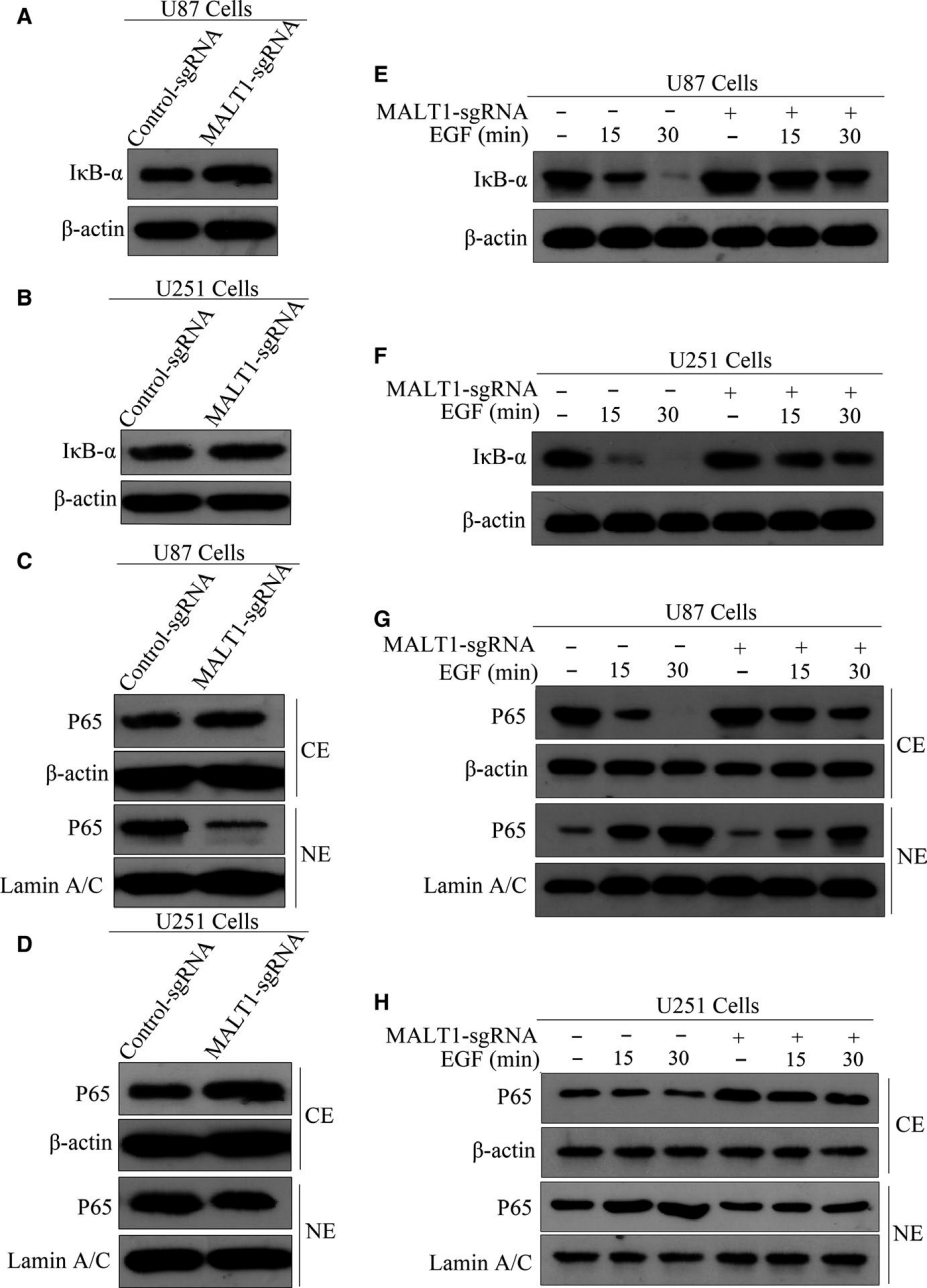
Knocking out MALT1 inhibits EGFR‐induced NF‐κB activation in GBM cells. (A and B) The effects of knocking out MALT1 on the levels of IκB‐α in U87 and U251 cells as assessed by Western blot analysis. (C and D) Subcellular location of p65 was detected using cellular fractionation and immunoblotting after knocking out MALT1. Lamin A/C and actin were used as nuclear and cytoplasm loading controls, respectively. (E and F) Knocking out MALT1 inhibited EGF‐induced degradation of IκB‐α. The cells were then stimulated with EGF (100 ng/mL) for the indicated times. IκB‐α expression was analysed by Western blot analysis. (G and H) MALT1 knockout suppressed EGF‐induced nuclear translocation of p65 in GBM cells. The cells were stimulated with EGF (100 ng/mL) for the indicated time, and then, cell lysates were subjected to Western blotting using p65 antibodies

To test whether MALT1 is involved in EGF‐induced NF‐κB activation, we examined the effects of MALT1 deficiency or inhibition on the activation of NF‐κB signalling. IκB‐α levels in MALT1‐knockout GBM cells were examined because IκB‐α degradation occurs before NF‐κB nuclear translocation. Knocking out MALT1 in U87 or U251 cells induced an increase in IκB‐α expression levels, as indicated by Western blot analysis (Figure 5A,B). In addition, knocking out MALT1 induced a significant decrease in nuclear p65 expression, whereas an increase in expression of cytoplasmic p65 was observed (Figure 5C,D). To test this finding, we applied EGF to stimulate the U87 and U251 cells and found that IκB‐α was degraded effectively. In addition, IκB‐α degradation was partially abolished when MALT1 was knocked out (Figure 5E,F). In particular, knocking out MALT1 abrogated EGF‐induced p65 nuclear localization upon EGF stimulation, which indicates that MALT1 expression improves EGF‐induced NF‐κB nuclear translocation (Figure 5G,H).

**FIGURE 6:**
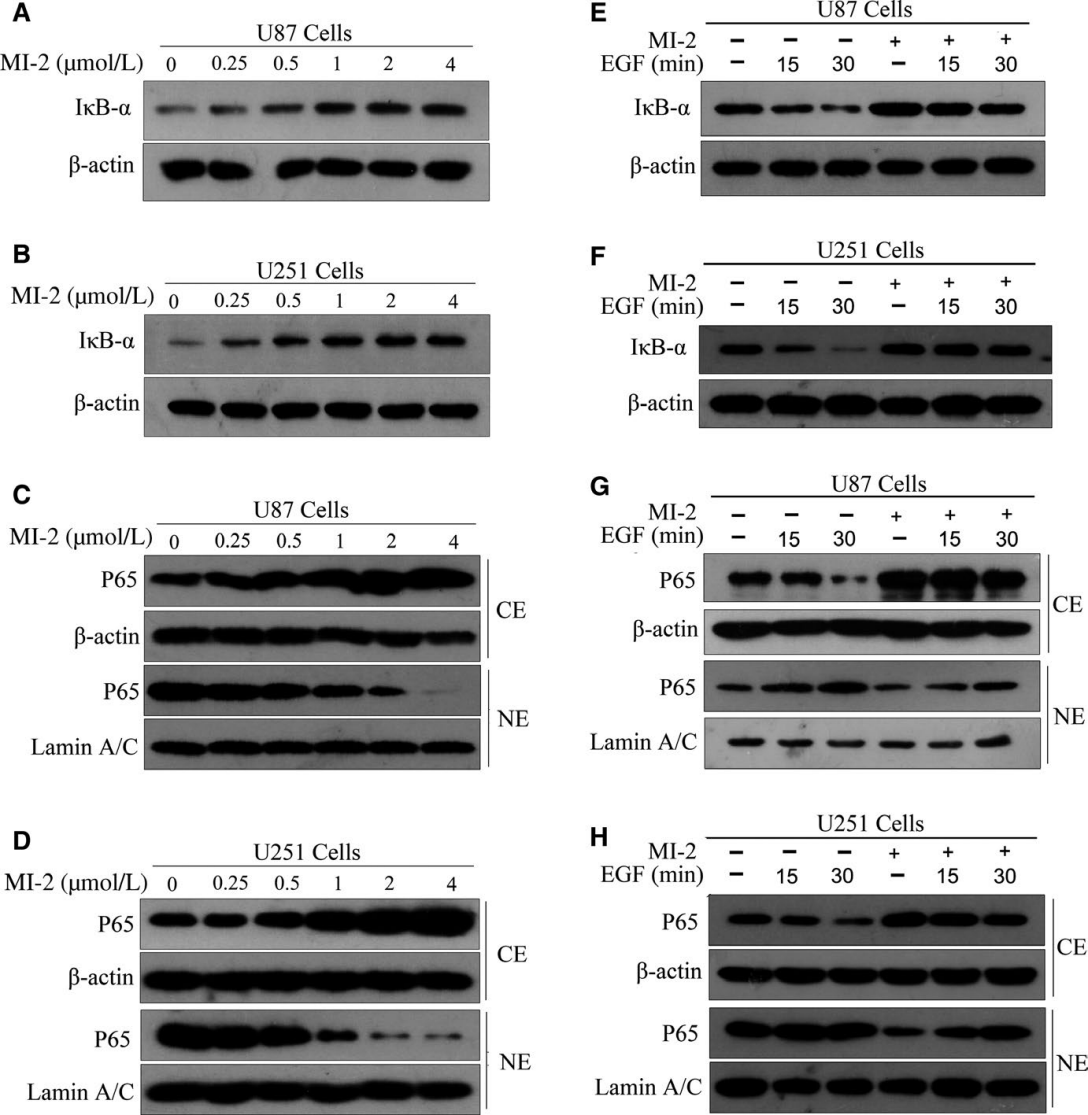
MI‐2 suppresses EGFR‐induced NF‐κB activation in GBM cells. (A and B) Cells were treated with MI‐2 at the indicated concentrations for 2 h. Total protein extracts isolated from U87 and U251 cell lines were evaluated using Western blotting assays with the anti‐IκB‐α antibody. (C and D) MI‐2 suppressed EGF‐induced degradation of IκB‐α. U87 and U251 cells were pre‐treated with or without MI‐2 (1 μmol/L) for 2 h. The cells were then incubated with EGF (100 ng/mL) for 15 or 30 min. The expression levels of IκB‐α were assessed by Western blotting. (E and F) MI‐2 inhibited nuclear accumulation of p65 in GBM cells. U87 and U251 cells were treated with vehicle or MI‐2 (0‐4 μmol/L) for 2 h. Cytoplasmic and nuclear protein extracts were analysed by immunoblotting with the indicated antibodies. (G and H) MI‐2 inhibited EGF‐induced nuclear translocation of p65 in GBM cells

MALT1 cells pre‐treated with MI‐2 were used to test its function in EGFR‐induced NF‐κB activation. The results suggested that MI‐2 treatment inhibited MALT1 effectively, increased IκB‐α expression and suppressed nuclear accumulation of the NF‐κB P65 subunit (Figure 6A‐D). Moreover, EGF‐treated GBM cells were induced to undergo IκB‐α degradation and nuclear accumulation of the NF‐κB P65 subunit. However, this was abolished by MI‐2 pre‐treatment (Figure 6E‐H). Taken together, these results reveal that MALT1 plays a key role in EGFR‐mediated NF‐κB activation in GBM cells.

## 4 DISCUSSION

GBMs are high‐grade gliomas that generally have poor prognosis because of intrinsic malignancy and drug/radiation resistance.^1^ Despite advances in surgical techniques, adjuvant radiotherapy and chemotherapy, there have been no valid strategies for curing GBM patients to date.^29^ Thus, the development of novel targeted therapeutics for GBM is urgently needed. In this study, inhibiting MALT1 levels using genetic and pharmacological approaches suppressed the growth of GBM cells in vitro and in vivo. Importantly, MALT1 is required for EGFR‐induced NF‐κB activation in GBM cells.

MALT1 is a well‐known effector protein in immune cells that regulates NF‐κB–driven adaptive immune responses.^15^ The up‐regulation of MALT1 is linked to the development of leukaemia and lymphoma.^30^, ^31^, ^32^ Therefore, MALT1 is currently considered as a promising therapeutic target for treating lymphomas and autoimmune disorders. Recent studies have shown that MALT1 as an oncogene is up‐regulated in various solid tumours and may be a key factor for tumour development.^33^, ^34^, ^35^, ^36^ A recent study showed that miR‐181d is down‐regulated in GBMs and affects the proliferation of GBM cells. Interestingly, MALT1 was identified as a direct target of miR‐181d, which could attenuate the mesenchymal phenotype by directly repressing MALT1 expression in glioblastoma.^19^ Our results show that knocking out MALT1 knockout results in the inhibition of GBM cell growth in vivo and in vitro, and the survival rate was increased accordingly. Pre‐clinical animal experiments have shown that MI‐2 is a MALT1 inhibitor that can avert lymphoma progression.^20^, ^37^ Similarly, we also found that MI‐2–treated mice inhibited the growth rate of intracerebral xenografted tumours. Taken together, our results illustrate that MALT1 works as a selective target of GBM, and MI‐2 may be used as an effective therapeutic agent for GBM.

Recent studies have reported that NF‐κB activation is very common in GBM and the underlying regulatory mechanisms have been elucidated.^38^, ^39^, ^40^ The amplification and mutation of the *EGFR* gene are among the key genetic alterations in GBM, and aberrant EGFR signalling is a key activator of NF‐κB activation in GBM.^41^ Notably, EGFR promotes the survival and chemotherapy resistance of GBM cells by Akt‐independent activation of the NF‐κB pathway.^14^ However, the contribution of NF‐κB to EGFR‐associated tumour progression remains unclear. Pan et al reported that MALT1 is required for EGFR‐induced NF‐κB activation in lung cancer cells. They found that MALT1 mainly functions as a scaffold protein that recruits TRAF6 to the IKK complex to activate NF‐κB after EGF stimulation. MALT1 deficiency suppresses EGFR‐induced NF‐κB activation.^18^ Consistent with their results, we also found that MALT1 knockout/inhibition significantly blocks IκB‐α degradation and abolishes nuclear accumulation of NF‐κB p65 subunit upon EGF stimulation in GBM cells. IκB‐α is a core controller of NF‐κB activation and is phosphorylated by IKK (IκB‐α kinase) proteins to release active NF‐κB.^42^, ^43^ NF‐κB can modulate a wide range of genes related to inflammation, apoptosis resistance, cell proliferation and cell invasion via binding with DNA.^43^ Hence, the nuclear accumulation of NF‐κB p65 subunit is significantly important in tumorigenesis and development of GBM. Our data further confirmed the important role of MALT1 in EGFR‐induced NF‐κB activation. These findings provide the genetic evidence that targeting MALT1 in NF‐κB signalling in EGFR‐associated GBMs. However, we did not investigate the mechanism underlying this phenomenon in in vivo studies. Further research is needed to identify the mechanism of MALT1 abnormal activation in GBM. In addition, MALT1 is also a protease. API2‐MALT1 fusion oncoprotein can cleave NF‐κB–inducing kinase (NIK), an essential mediator of non‐canonical NF‐κB signalling that regulates non‐canonical NF‐κB signalling.^44^ Moreover, Wang et al reported that MALT1 promotes melanoma progression via JNK/c‐Jun signalling.^34^ Therefore, whether MALT1 is also involved in regulating non‐canonical NF‐κB signalling or other signalling pathways in glioma cells should be confirmed in future studies.

This is the first systematic study on the role of MALT1 in GBM progression. We found that MALT1 plays critical roles in cell proliferation in vitro and in vivo by inducing G1 cell cycle arrest. Most importantly, we further confirmed that EGFR‐induced NF‐κB activation can be significantly blocked by MALT1 inhibition and further regulate GBM cell proliferation and survival. These findings suggest that MALT1 is a potential theoretical target for GBMs and provide a hint for clinical trials to evaluate the therapeutic potential of MALT1 inhibitor in human GBMs.

## CONFLICT OF INTEREST

The authors declare that they have no competing interests.

## AUTHOR CONTRIBUTIONS

MN and RY designed this study. XL, CY and LS performed the main experimental procedures. GL, QC, QS, YW, XC and HL carried out partial experiments. JW and SG performed the statistical analysis. XL and MN wrote this manuscript. All the authors read and approved the final manuscript.

## Data Availability

The data sets supporting the conclusions of this article are included in the article.
