# CORRIGENDUM

**DOI:** 10.1111/jcmm.17302

**Published:** 2022-05-07

In Xuejiao Liu et al.,^1^ the published article contains errors in Figure 5C,D. The P65 bands are incorrect in the original publication. The corrected Figure 5 is shown below. The authors confirm that all the results and conclusions of this article remain unchanged.

**FIGURE 5:**
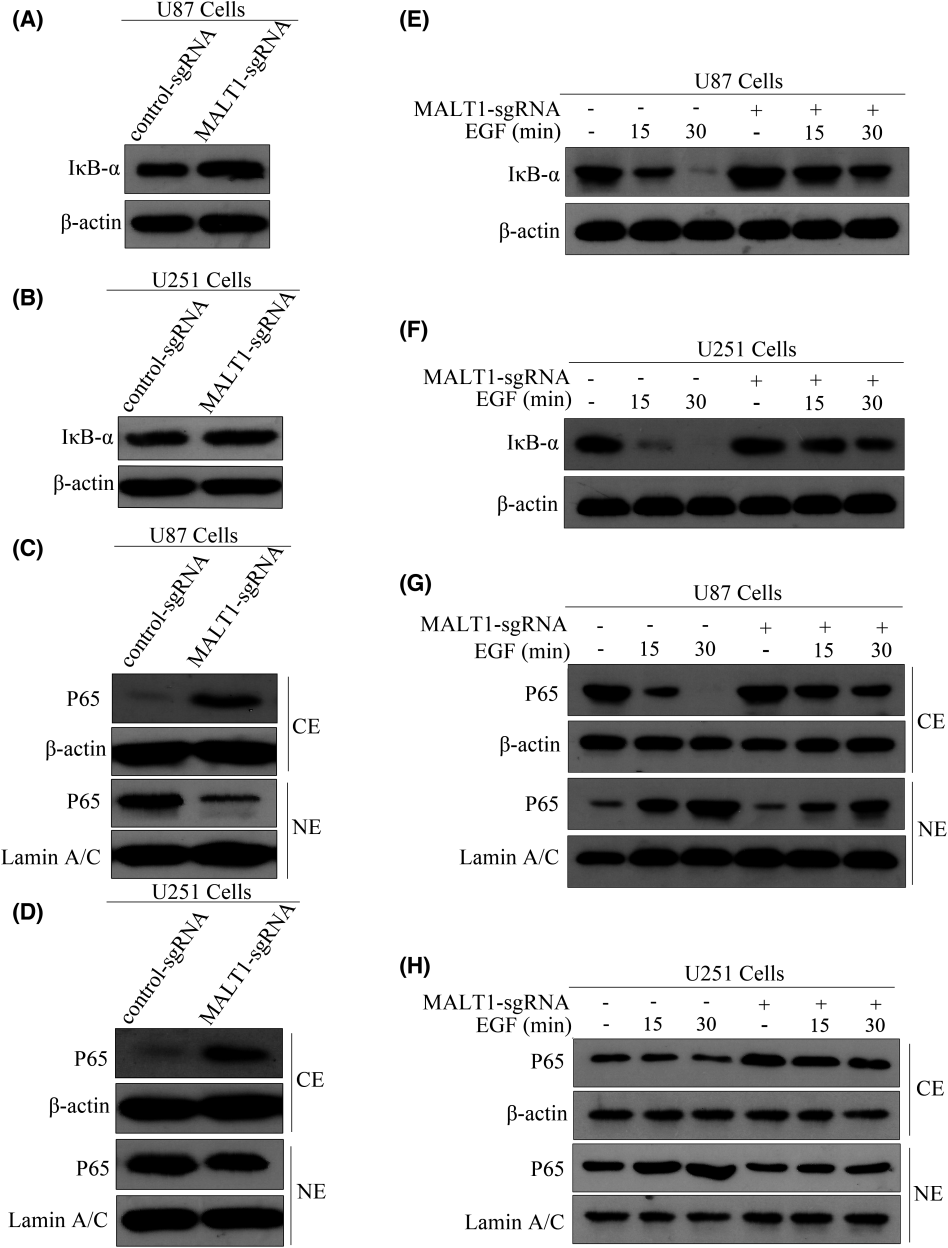
Knocking out MALT1 inhibits EGFR‐induced NF‐κB activation in GBM cells. (A and B) The effects of knocking out MALT1 on the levels of IκB‐α in U87 and U251 cells as assessed by western blot analysis. (C and D) Subcellular location of p65 was detected using cellular fractionation and immunoblotting after knocking out MALT1. Lamin A/C and actin were used as nuclear and cytoplasm loading controls, respectively. (E and F) Knocking out MALT1 inhibited EGF‐induced degradation of IκB‐α. The cells were then stimulated with EGF (100 ng/mL) for the indicated times. IκB‐α expression was analysed by western blot analysis. (G and H) MALT1 knockout suppressed EGF‐induced nuclear translocation of p65 in GBM cells. The cells were stimulated with EGF (100 ng/mL) for the indicated time, and then cell lysates were subjected to western blotting using p65 antibodies.
